# Acute Effects of Caffeine on Choroidal Structure and Vascularity in Healthy Young Adults: A Time-Resolved OCT and OCT-Angiography Study

**DOI:** 10.3390/nu18203426

**Published:** 2026-10-19

**Authors:** Eleonora D’Aniello, Livio Vitiello, Maddalena De Bernardo, Deokho Lee, Ilaria De Pascale, Gerardo La Padula, Stefano Gallo, Alfonso Pellegrino, Nicola Rosa

**Affiliations:** 1 Eye Unit, “Luigi Curto” Hospital, Azienda Sanitaria Locale Salerno, 84035 Polla, Italy; danielloeleonora@gmail.com (E.D.); idepascale@outlook.it (I.D.P.); al.pellegrino@aslsalerno.it (A.P.); 2 Department of Medicine, Surgery and Dentistry “Scuola Medica Salernitana”, University of Salerno, 84081 Baronissi, Italy; mdebernardo@unisa.it (M.D.B.); gerardo.lp@outlook.it (G.L.P.); stefano.gallo156@gmail.com (S.G.); nrosa@unisa.it (N.R.); 3 The Korean Institute of Nutrition, Hallym University, 1 Hallymdeahak-Gil, Chuncheon 24252, Republic of Korea

**Keywords:** caffeine, choroid, choroidal thickness, choroidal vascularity index, intraocular pressure, nutrition and eye health, ocular vascular aging, optic nerve head

## Abstract

**Background**: Caffeine, the most widely consumed dietary bioactive compound worldwide, exerts acute vasoactive effects on several vascular beds, but its impact on the internal composition of the choroid remains unclear. We evaluated the acute effects of a single oral dose of caffeine on choroidal structure and vascularity, retinal superficial capillary plexus (SCP) microcirculation, optic nerve head (ONH) perfusion, and intraocular pressure (IOP) in healthy young adults. **Methods**: In this prospective, single-arm observational study without a control group, 46 healthy adults were examined at baseline and 30 and 60 min after ingesting 200 mg of caffeine. Subfoveal choroidal thickness (SCT), total, luminal and stromal choroidal areas, and the choroidal vascularity index (CVI) were derived from EDI-OCT with Niblack binarization. SCP vessel density and perfusion, ONH perfusion and flow index, and IOP were also recorded. Data were analyzed using repeated-measures ANOVA. **Results**: SCT decreased progressively from 356.0 ± 84.2 µm to 325.9 ± 81.0 µm at 60 min (*p* < 0.001; η^2^*p* = 0.61). Luminal and stromal areas declined in parallel (r = 0.919, *p* < 0.001), with only a minimal change in CVI (*p* = 0.003). IOP decreased modestly (*p* = 0.009), whereas SCP and ONH parameters showed no significant changes. The response was independent of sex and axial length. **Conclusions**: A single dose of caffeine was associated with acute, progressive choroidal thinning, paralleled by a near-proportional constriction of the luminal and stromal compartments, with no significant short-term change detected in the retinal and optic nerve head circulations, consistent with an adenosine-mediated vasoconstrictive mechanism.

## 1. Introduction

Caffeine is the most widely consumed dietary bioactive compound in the world, ingested daily through coffee, tea, chocolate and a range of beverages [1]. Beyond its role as a stimulant, caffeine is increasingly recognized as a dietary component with relevant effects on ocular physiology: experimental evidence attributes to caffeine and to adenosine A2A-receptor antagonists anti-inflammatory [2], anti-angiogenic [3] and potentially neuroprotective properties [4] in retinal and choroidal tissue, while acute intake exerts measurable hemodynamic effects on the ocular circulation [5]. Because the choroid and the retina are among the most metabolically active and vascular-dependent tissues of the human body, understanding how a widely consumed dietary compound acutely modulates their perfusion is of direct relevance to ocular vascular health and its age-related deterioration. Given how frequently caffeine is consumed, even a transient effect on the ocular circulation may act as an unrecognized confounder in imaging-based studies of the choroid and retina, and may carry implications for the interpretation of vascular biomarkers in both research and clinical settings.

Caffeine is a non-selective antagonist of the adenosine receptors (A1, A2A, A2B and A3), which are widely distributed in the central nervous system and peripheral tissues [6,7]. Under physiological conditions adenosine exerts a diffuse inhibitory effect and promotes vasodilation through A2A and A2B activation; competitive antagonism by caffeine therefore increases neuronal activity, sympathetic tone and arterial pressure [8]. In healthy subjects, coffee acutely increases muscle sympathetic nerve activity and blood pressure, effects that are more pronounced in non-habitual consumers, suggesting pharmacodynamic tolerance in chronic users [9,10].

Caffeine also modulates cerebral vasoregulation: acute administration can reduce cerebral blood flow by 20–30%, consistent with an A2A-mediated vasoconstrictor effect, while overall cerebral perfusion is preserved through autoregulation [11,12,13]. These properties underlie its use as an analgesic adjuvant in combination formulations for migraine and tension-type headache, where the adenosine-mediated cerebral vasoconstriction counteracts the meningeal vasodilation of the migrainous phase [14,15,16].

Several studies have investigated the acute ocular effects of caffeine using optical coherence tomography (OCT) and OCT angiography (OCTA). A significant reduction in subfoveal and peripapillary choroidal thickness has been reported [17,18,19,20], more evident in myopic subjects [21]. OCTA studies have also described transient reductions in superficial capillary plexus (SCP) vessel density, absent after decaffeinated coffee and reversible within one hour [22,23], as well as changes in the deep capillary plexus [24] and a delay in retinal neurovascular coupling [25]. The effect of caffeine on intraocular pressure (IOP) has also been studied, with a mild increase in healthy subjects and a more marked rise in glaucoma patients or non-habitual consumers [26,27]. Experimental work additionally suggests long-term neuroprotective, anti-inflammatory and anti-angiogenic effects in ischemic and degenerative retinal disease [2,3,28].

The recent literature on these topics supports a dual effect of caffeine: acute vasoconstriction and chronic protection, dependent on dose, frequency and pathophysiological context [2,3,14,15,16,28]. However, three gaps persist. First, most studies have assessed single ocular parameters in isolation, precluding any judgment on whether caffeine acts selectively on one vascular structure or globally on the eye [17,19]. Second, although choroidal thinning is well documented, the caffeine-induced changes inside the choroidal structure have rarely been quantified, and the few studies on the choroidal vascularity index (CVI) remain scarce and discordant [20]. Third, the optic nerve head (ONH) circulation has not been examined in this context, despite its clinical relevance given the widespread use of caffeine in combination analgesics for migraine. Because choroidal thickness and perfusion physiologically decline with age [29,30], characterizing how a common dietary compound acutely modulates the choroidal vascular compartments may also inform our understanding of ocular vascular aging.

The present study was designed to fill these gaps, allowing us to move beyond merely confirming the choroidal thinning: to test how it occurs (compartmental analysis), where the effect is anatomically confined (choroid versus retina versus ONH), and whether the CVI is genuinely preserved or merely appears so.

## 2. Materials and Methods

### 2.1. Study Design and Participants

This single-center prospective, single-arm observational study (without a placebo or control group) was conducted between July and September 2025 at the Eye Unit of “Luigi Curto” Hospital (Azienda Sanitaria Locale Salerno, Polla, Italy) in a homogeneous cohort of healthy young volunteers, evaluating caffeine-induced changes on subfoveal choroidal thickness (SCT), total, luminal and stromal choroidal areas, CVI, retinal SCP density and perfusion, ONH perfusion and flow index, and IOP. OCT acquisitions were performed within a fixed time window in the morning (10:00–12:00) to minimize diurnal variability [31]. Only the right eye of each participant was evaluated. Forty-six healthy volunteers were enrolled. Inclusion criteria were: age 20–40 years; abstinence from caffeine for at least 24 h before examination; axial length 21.0–26.5 mm; refractive error ≤ 5 D (sphere) and ≤3 D (cylinder); and OCT/OCTA image quality > 7/10. Exclusion criteria were: amblyopia; previous ocular surgery; systemic or ocular disease; smoking [32]; caffeine intolerance or failure to abstain from caffeine for at least 24 h; and pregnancy.

All participants underwent a complete ophthalmological examination to exclude ocular or systemic disease, including best-corrected visual acuity, subjective and objective refraction, anterior-segment biomicroscopy and dilated fundus examination. Axial length was measured by optical interferometric biometry (IOL Master 700, Zeiss, Jena, Germany) to exclude significant anatomical differences between subjects.

As all participants received caffeine and no placebo or control group was included, the observed temporal changes should be interpreted as associated with caffeine intake rather than being attributable to it with the certainty afforded by a controlled or crossover design.

The study followed the tenets of the Declaration of Helsinki, and written informed consent was obtained from all participants, together with Institutional Review Board approval, obtained under an institutional framework protocol for the observational collection and analysis of ophthalmic imaging data that remains active and encompasses the present study (ComEtico Campania Sud, prot. n 16544, date of approval: 8 March 2017).

### 2.2. Experimental Protocol

Each participant followed a standardized protocol comprising three time points: baseline measurements (T0), followed by oral administration of a single tablet containing exclusively 200 mg of pure caffeine, identical for all participants, and two follow-up assessments at 30 and 60 min after intake. The same evaluations were repeated at each session: IOP measurement, Enhanced Depth Imaging (EDI) OCT acquisition for choroidal parameters (SCT and CVI-related areas) [30], macular 6 × 6 mm OCTA for SCP density and perfusion, and ONH OCTA (4.5 × 4.5 mm) for ONH perfusion and flow index. IOP was measured with non-contact tonometry (Keeler Pulsair IntelliPuff, Keeler Ltd., Windsor, UK), with the subject seated and after a few minutes of rest to stabilize heart rate and systemic pressure.

### 2.3. Choroidal Imaging and Image Analysis

**Figure 1:**
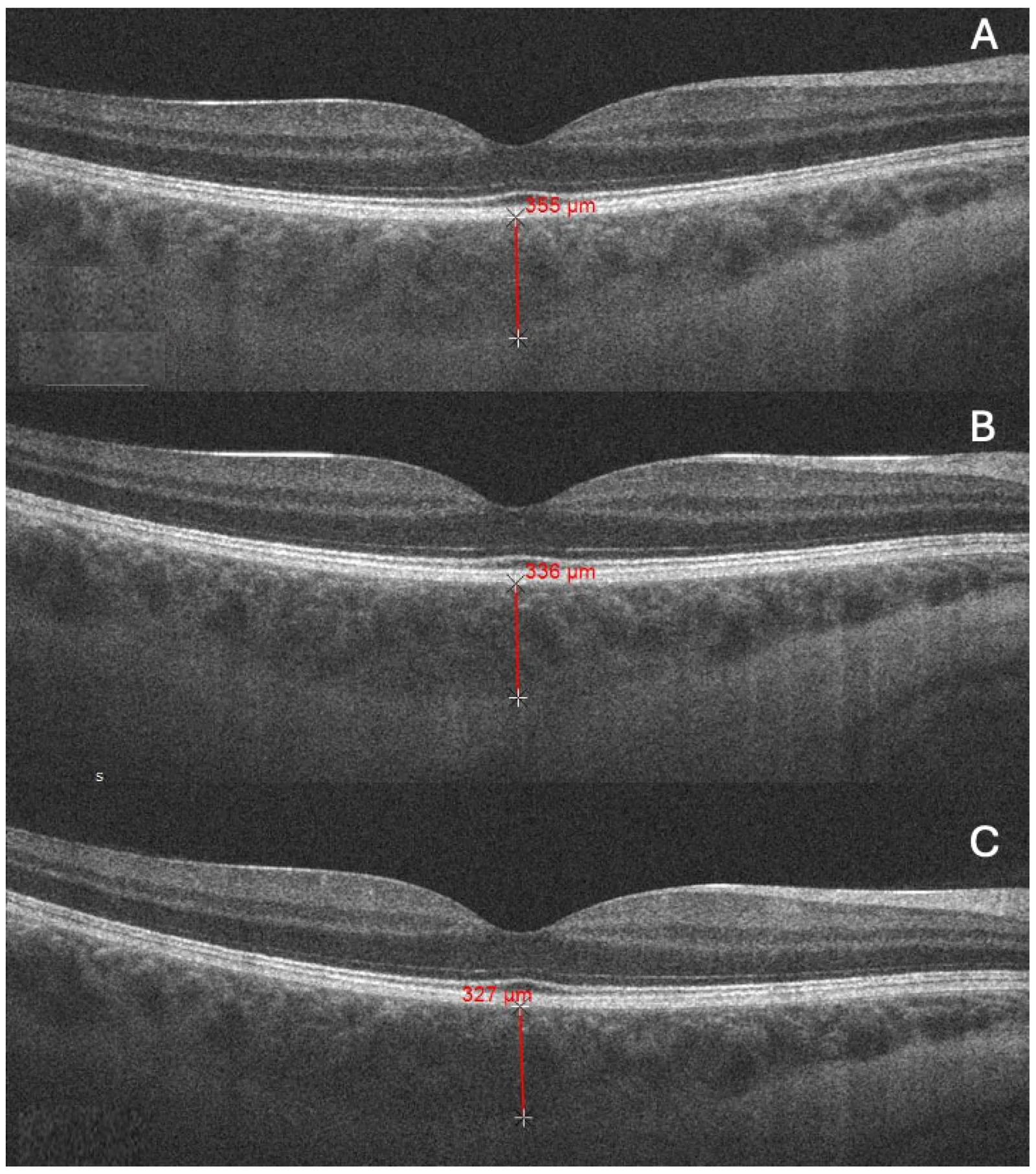
Serial enhanced depth imaging OCT (EDI-OCT) scans of a representative subject at baseline (T0, (**A**)), 30 min (**B**), and 60 min (**C**) after caffeine intake, showing the progressive reduction in SCT, measured as the perpendicular distance between the RPE and the choroidal–scleral interface. Measured values: T0 = 355 µm; 30 min = 336 µm; 60 min = 327 µm.

OCT acquisitions were performed with a Cirrus 5000 Angioplex (Carl Zeiss Meditec, Dublin, CA, USA) in HD 5-line raster EDI mode, centered on the fovea. SCT was measured manually as the perpendicular distance between the outer border of the retinal pigment epithelium (RPE) and the inner border of the choroidal–scleral interface (CSI), according to Margolis and Spaide (Figure 1) [30].

All measurements were performed by a single experienced observer (E.D.) under constant illumination to avoid the blooming effect [33] and with signal-quality control, to maximize intra-observer reproducibility.

**Figure 2:**
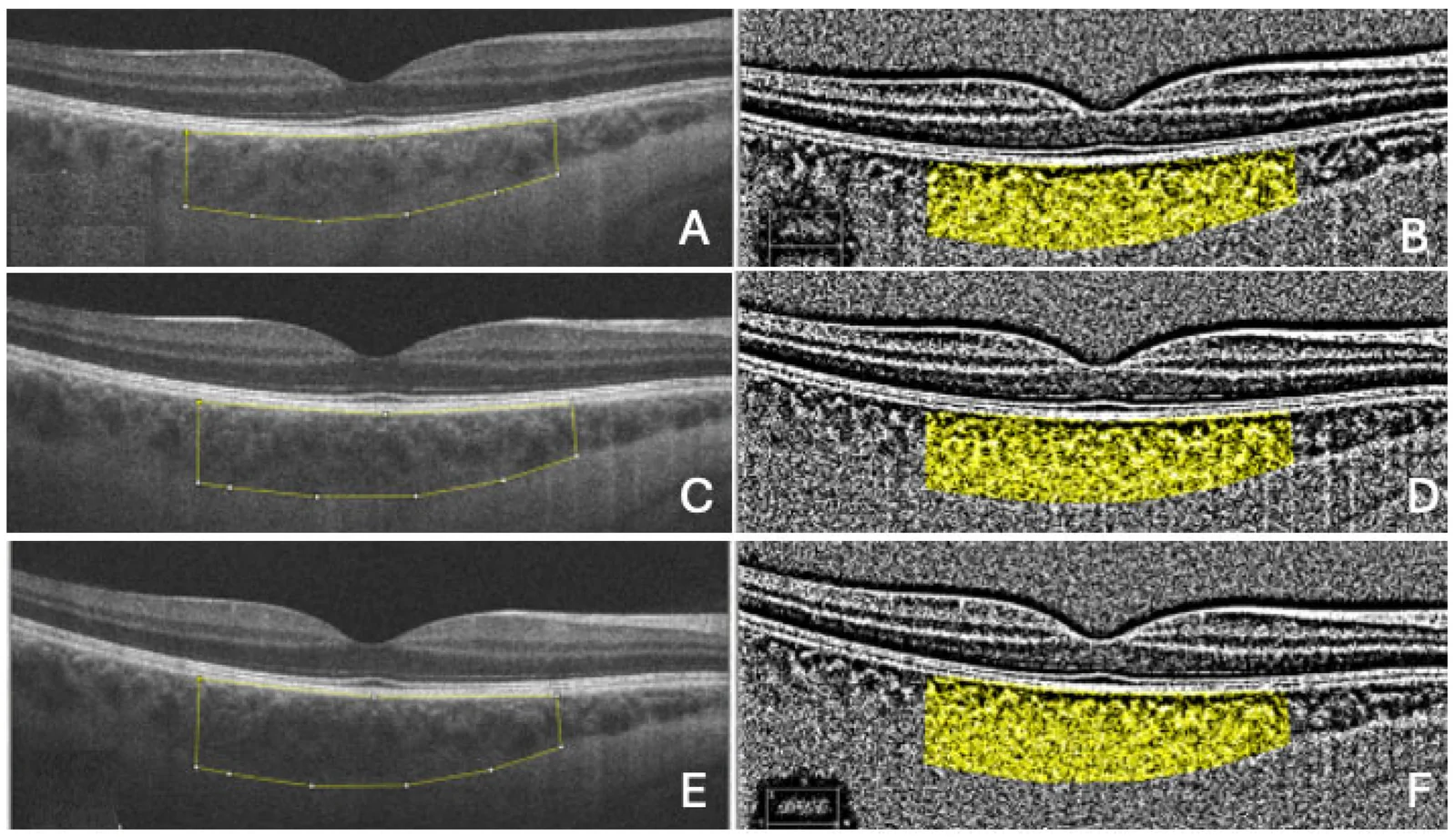
Serial choroidal binarization in ImageJ (Niblack method) of the same representative subject at the three time points (T0—(**A**,**B**); 30 min—(**C**,**D**); 60 min—(**E**,**F**)), according to Agrawal et al.

Total (TCA), luminal (LCA) and stromal (SCA) choroidal areas were processed in ImageJ (v1.52) following the protocol of Agrawal et al. [34]. Given the pixel anisotropy of the Cirrus device, the scale was set globally to 6.40 µm/pixel horizontally and 3.20 µm/pixel vertically (aspect ratio 0.5). For each scan, a polygonal region of interest (ROI) was defined between the RPE interface and the CSI, extending ±1500 µm around the fovea; the same ROI was applied at the three time points, adjusting only the inferior CSI margin to preserve intra-eye comparability. Images were binarized using the Niblack threshold and converted to RGB to separate dark pixels (luminal component, LCA) from light pixels (stromal component, SCA). TCA corresponds to the ROI; SCA was obtained by subtraction (SCA = TCA − LCA); and the CVI was calculated as LCA/TCA (Figure 2).

### 2.4. Retinal and Optic Nerve Head OCT-Angiography

Retinal and ONH microvascular assessments were performed using OCTA, a non-invasive, dye-free imaging technique that does not require any intravenous contrast. Macular 6 × 6 mm OCTA restricted to the SCP was used. All images were acquired with automatic segmentation, checked for signal strength ≥ 7/10 and exported for quantitative analysis. Two parameters were computed: vessel density, expressed as the ratio between total vessel length and the analyzed area (mm/mm^2^), and perfusion density, expressed as the percentage of retinal area with detectable capillary flow after binarization.

In addition, ONH microcirculation was assessed with the same device using the 4.5 × 4.5 mm ONH angiography module, from which ONH perfusion and the ONH flow index were derived.

### 2.5. Statistical Analysis

All analyses were performed with Jamovi software (version 2.7.34) and G*Power software (version 3.1.9.7) [35]. Continuous variables are reported as mean ± standard deviation. For each parameter, the effect of caffeine across the three time points was assessed with one-way repeated-measures analysis of variance (RM-ANOVA), with time as the within-subject factor.

Normality was checked with the Shapiro–Wilk test and sphericity with Mauchly’s test; the Greenhouse–Geisser correction was applied when sphericity was violated. Where normality was not fully met, results were cross-validated with the Friedman test, which confirmed the parametric findings in all cases. Effect size was quantified as partial eta-squared (η^2^*p*). Significant omnibus tests were followed by Holm-corrected pairwise comparisons. Individual changes (Δ = 60 min − baseline) of the choroidal parameters were also correlated using Pearson’s coefficient to explore the underlying mechanism; sex was added as a between-subject factor for the two primary outcomes (SCT and CVI), and axial length was also correlated with the Δ of the primary outcomes.

An a priori power analysis (three measurements, α = 0.05, power = 0.90), based on the prespecified CVI effect size (Cohen’s f = 0.37), indicated that 16 participants were required; the final sample of 46 exceeded this target. A two-tailed *p* < 0.05 was considered significant.

## 3. Results

### 3.1. Study Population

Forty-six healthy volunteers (18 men, 28 women) were analyzed. Mean age was 28.3 ± 5.0 years (range 20–39) and mean axial length was 24.5 ± 1.2 mm (range 21.1–26.4). Descriptive statistics for all parameters at the three time points are summarized in Table 1, while the results of the RM-ANOVA for all parameters are reported in Table 2.

### 3.2. Choroidal Thickness

SCT showed the strongest response, decreasing progressively from 356.0 ± 84.2 µm at baseline to 339.7 ± 82.3 µm at 30 min and 325.9 ± 81.0 µm at 60 min (F(1.30, 58.7) = 70.6, *p* < 0.001, Greenhouse–Geisser corrected; η^2^*p* = 0.61). All pairwise comparisons were significant (all Holm-adjusted *p* < 0.001), including the 30-to-60 min comparison, indicating that thinning continued throughout the observation period (overall reduction of about 8.5%).

### 3.3. Choroidal Areas

All area components decreased significantly. TCA declined from 1.048 ± 0.236 mm^2^ to 0.995 ± 0.212 mm^2^ (F(1.43, 64.4) = 20.0, *p* < 0.001; η^2^*p* = 0.31); SCA from 0.390 ± 0.097 mm^2^ to 0.366 ± 0.089 mm^2^ (F(1.58, 71.0) = 19.8, *p* < 0.001; η^2^*p* = 0.31); and LCA from 0.659 ± 0.140 mm^2^ to 0.629 ± 0.123 mm^2^ (F(1.47, 66.0) = 17.8, *p* < 0.001; η^2^*p* = 0.28). Every pairwise comparison was significant (Holm-adjusted *p* ≤ 0.001). Because sphericity was violated, Greenhouse–Geisser–corrected values are reported; the Friedman test confirmed all results (all *p* < 0.001). Pairwise comparisons for all significant parameters are detailed in Table 3.

**Figure 3:**
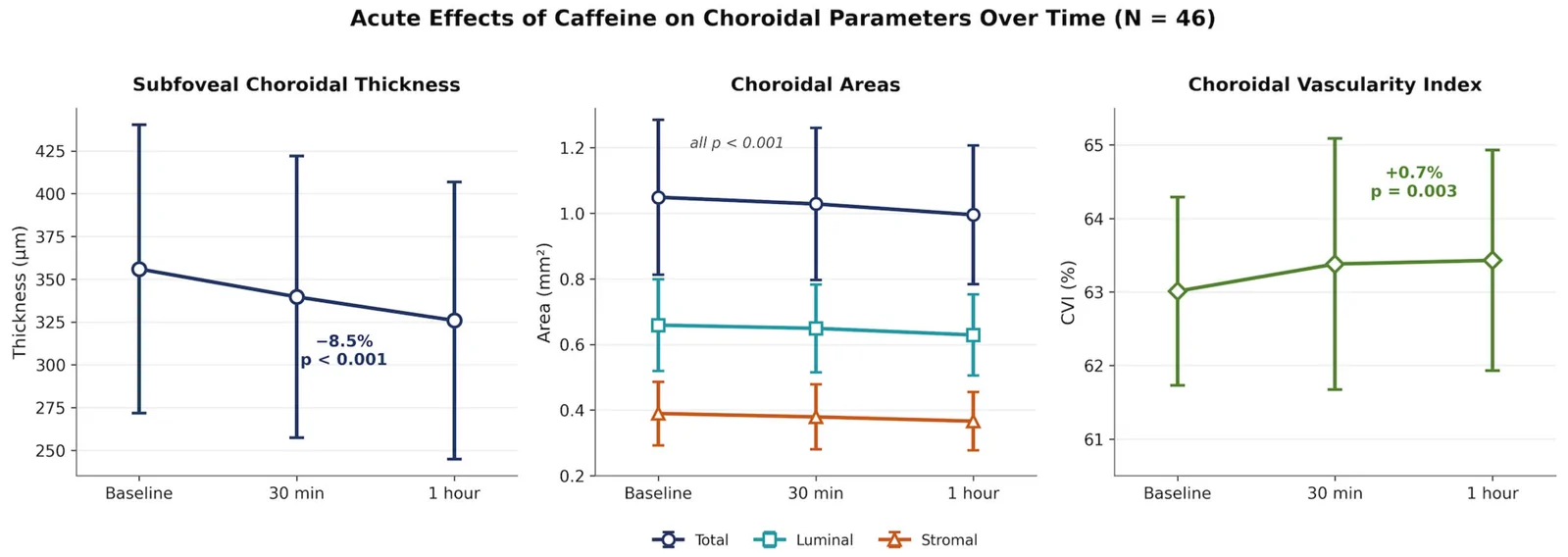
Time course of the main choroidal parameters after a single 200 mg oral dose of caffeine (n = 46). Subfoveal choroidal thickness (**left**), total, luminal and stromal choroidal areas (**center**), and the choroidal vascularity index (**right**) at baseline, 30 and 60 min. Data are presented as mean ± standard deviation. Choroidal thickness declined progressively across all time points, whereas the choroidal vascularity index showed only a minimal increase that stabilized after 30 min.

Conversely, the CVI showed a small but significant increase, from 63.01 ± 1.28% at baseline to 63.38 ± 1.71% at 30 min and 63.43 ± 1.50% at 60 min (F(2, 90) = 6.13, *p* = 0.003; η^2^*p* = 0.12). The increase was significant from baseline to 30 min (*p* = 0.036) and to 60 min (*p* = 0.006), but not between 30 and 60 min (*p* = 0.653). The time course of the main choroidal parameters is shown in Figure 3.

### 3.4. Intraocular Pressure

IOP decreased from 13.9 ± 2.2 mmHg to 13.1 ± 2.3 mmHg at 30 min and 13.0 ± 2.7 mmHg at 60 min (F(1.65, 74.3) = 5.52, *p* = 0.009; η^2^*p* = 0.11), significant from baseline to both follow-ups but not between 30 and 60 min (*p* = 0.857).

### 3.5. Retinal and Optic Nerve Head Microcirculation

No significant time effect was observed for SCP vessel density (F(2, 90) = 0.90, *p* = 0.411), SCP perfusion (F(2, 90) = 0.68, *p* = 0.508), ONH perfusion (F(2, 90) = 1.21, *p* = 0.303) or ONH flow index (F(2, 90) = 1.31, *p* = 0.275). Thus, the observed response was predominantly choroidal, with no significant changes detected in the retinal SCP or ONH vascular parameters. Notably, the study was powered for the CVI rather than for these parameters, so their non-significance should be interpreted as the absence of a detectable short-term effect rather than as evidence of equivalence.

### 3.6. Mechanism, Sex and Axial Length

The reductions in LCA and SCA were strongly and positively correlated (r = 0.919, *p* < 0.001), indicating that both compartments contracted concomitantly; however, the stromal area decreased to a proportionally greater extent than the luminal area, which accounts for the slight increase observed in the CVI. The change in CVI correlated moderately with the change in stromal area (r = −0.539, *p* < 0.001), whereas it was not significantly associated with the change in luminal area (r = −0.186, p = 0.216), indicating that the slight increase in CVI was driven by the proportionally greater reduction of the stromal compartment, whereas the change in SCT was unrelated with the change in SCA (r = −0.026, *p* = 0.865) or LCA (r = −0.108, *p* = 0.476), consistent with thickness and area capturing geometrically distinct dimensions. No significant time × sex interaction was found for SCT (*p* = 0.63) or CVI (*p* = 0.27), and axial length was not correlated with the magnitude of change in SCT (r = 0.210, *p* = 0.16) or CVI (r = −0.092, *p* = 0.55). Given the moderate sex imbalance (18 men, 28 women), the absence of an interaction should be read as no detectable difference rather than proof of equivalence.

## 4. Discussion

In this study, we used a combined OCT and OCTA protocol to characterize, in a time-resolved manner, the acute ocular effects of a single 200 mg oral dose of caffeine in healthy young adults. By acquiring choroidal structural parameters (SCT, TCA, LCA and SCA), the CVI, retinal SCP density and perfusion, ONH perfusion and flow index, and IOP at the same three time points, we were able to relate structural and microvascular changes to one another rather than examining them in isolation. Three main findings stand out: the choroid thinned markedly and progressively; this thinning reflected parallel reductions in luminal and stromal choroidal areas; and the effect was anatomically selective, with no significant short-term change in the retinal SCP and the ONH circulation, while inducing a small reduction in IOP.

### 4.1. Choroidal Thinning as the Dominant Response

SCT showed by far the largest effect (η^2^*p* = 0.61), decreasing by approximately 8.5% within one hour. Importantly, the reduction was progressive: all pairwise comparisons were significant, including the 30-to-60 min step, indicating that the choroid was still thinning at the end of our observation window. This is consistent with previous reports of caffeine- or coffee-induced choroidal thinning by Altinkaynak [17], Dervişoğulları [18] and Koçak [20], and it reinforces the notion that the choroid, which is densely vascularized and highly responsive to vasoactive stimuli, is the ocular compartment most sensitive to the acute action of caffeine. The most parsimonious explanation is a mechanism consistent with adenosine-mediated vasoconstriction: by antagonizing A2A and A2B receptors, caffeine removes a tonic vasodilatory input, reducing luminal volume and, consequently, overall choroidal thickness. The parallel, modest decrease in IOP that we observed (from 13.9 to 13.0 mmHg) could be compatible with a small reduction in ocular volume load and does not, in isolation, carry clinical relevance in healthy eyes.

### 4.2. Concurrent Luminal and Stromal Constriction

The central mechanical contribution of this study concerns the internal composition of the choroid. All three area components (TCA, LCA and SCA) decreased significantly, and the individual changes (Δ) in LCA and SCA were very strongly correlated (r = 0.919, *p* < 0.001). In relative terms, at 60 min the luminal area decreased by 4.6% and the stromal area by 6.0% compared with baseline, indicating reductions in similar magnitude, with a slight stromal predominance. Together with the strong correlation between the individual changes in the two compartments (r = 0.919), this indicates that luminal and stromal areas contracted concordantly and to a broadly comparable extent. In other words, the two compartments did not change independently: they contracted together, although to a slightly different extent. This has a direct and initially counter-intuitive consequence for the CVI.

Because the CVI is defined as the luminal-to-total area ratio, the fact that the stromal compartment decreased proportionally more than the luminal one caused the ratio to increase slightly rather than remaining unchanged: as the total area declined mainly at the expense of the stroma, the relative contribution of the luminal component, and therefore the CVI, rose marginally. In our cohort the CVI increased significantly, but the effect was small (η^2^*p* = 0.12) and stabilized early, with no further change between 30 and 60 min.

This nuance is relevant when our results are placed alongside those of Koçak et al. [20], who reported reductions in total and luminal choroidal area without a significant change in CVI. In our larger cohort, the CVI showed a small but statistically significant increase, reflecting the proportionally greater reduction of the stromal compartment. Rather than contradicting the earlier report, our data refine it: the caffeine-induced choroidal constriction involves both compartments but affects the stroma to a slightly greater extent, so that the luminal-to-total ratio is marginally increased rather than preserved. The strong Δ-luminal/Δ-stromal correlation provides the quantitative basis for this interpretation, which a simple omnibus test on the CVI alone cannot identify. This also illustrates a broader methodological point: with adequate statistical power, a parameter may reach significance while remaining biologically modest, and effect sizes and mechanistic correlations, not *p*-values alone, should guide interpretation.

It is also worth noting that the change in SCT was not related to the change in SCA (r = −0.026) or LCA (r = −0.108). This is expected rather than contradictory: SCT is a single perpendicular linear measurement, whereas the areas are integrated across a ±1500 µm horizontal extent of the scan. Therefore, the two parameters geometrically capture distinct dimensions of the same tissue, and their independence at the individual level does not undermine the concordant group-level direction of change.

### 4.3. Selectivity for the Choroid

A further strength of the integrated design is the demonstration of anatomical selectivity. Neither SCP vessel density nor SCP perfusion changed significantly over time, indicating no detectable short-term change in macular capillary perfusion. This apparent stability of the retinal microcirculation, despite a systemic vasoconstrictive stimulus, is consistent with efficient retinal autoregulation, which buffers perfusion pressure changes to maintain a stable metabolic supply. Our OCTA findings are therefore closer to those of Jacobs et al. [23], who reported no relevant change, than to studies describing transient reductions in vessel density [19,22]; the heterogeneity across the published literature is most plausibly explained by differences in dose, caffeine matrix, timing, device and population.

By extending the protocol to the ONH, we were able to show that ONH perfusion and flow index likewise showed no significant short-term change. This extension addresses a specific gap identified in the original study design and is clinically pertinent: considering that caffeine is a common component of combination analgesics for migraine [13,14,15,36], and taking into account that migraine is itself a putative risk factor for glaucoma [37,38] through vasospasm and perfusion dysregulation [39], the demonstration that a standard caffeine dose does not acutely compromise ONH perfusion in healthy eyes is a reassuring baseline observation against which future studies in at-risk populations can be compared.

### 4.4. Clinical Implications

In healthy young eyes, the described effects were acute and of limited direct clinical relevance. Their importance is twofold. First, they are methodological: the timing of caffeine intake is a genuine confounder in any study or clinical assessment that relies on choroidal thickness or choroidal vascularity, because caffeine is associated with a rapid and progressive choroidal thinning of the order of 8–9% within one hour. Standardizing, or at least recording, recent caffeine consumption should be considered good practice in choroid-driven imaging protocols.

Second, beyond healthy eyes, our findings may have pathophysiological relevance in certain conditions, although these considerations remain hypothetical and require dedicated investigation in the relevant patient populations, since they cannot be directly inferred from a healthy cohort. In pachychoroid spectrum disorders and central serous chorioretinopathy, a diffuse choroidal vasoconstriction with a possible decongestant effect is mechanistically plausible, although whether it confers clinical benefit or, conversely, destabilizes the choroidal–retinal equilibrium remains unknown [2,3,28]. In age-related macular degeneration, experimental data suggest anti-angiogenic [28] and anti-inflammatory [2] actions of caffeine and A2A antagonists, but the evidence remains preclinical, and an excessive reduction in choroidal flow could be detrimental in eyes with compromised vascular reserve. It should be emphasized that angiogenic and inflammatory pathways were not assessed in the present study; they are discussed here only to contextualize our structural and vascular findings within the broader experimental literature.

In glaucoma and ocular hypertension, and in non-habitual consumers, even small hemodynamic or pressure changes may be more consequential than in healthy subjects, warranting caution in the timing of caffeine intake relative to diagnostic testing or IOP-lowering therapy [26,27]. Overall, these disease-specific scenarios should be regarded as hypotheses to be tested in dedicated studies on the corresponding patient populations, rather than as direct clinical implications of the present findings in healthy young adults.

Framed within the broader context of nutrition and ocular aging, these findings illustrate how a single dietary compound can acutely reshape choroidal hemodynamics; whether habitual dietary caffeine intake exerts cumulative effects on the aging choroid remains an open question that dietary-interventional studies should address.

### 4.5. Strengths and Limitations

The main strengths of this study are the standardization of acquisition conditions (fixed morning time window, controlled environment, single experienced operator), the within-subject repeated-measures design that minimizes inter-individual variability and increases statistical power, the reproducible Niblack binarization protocol for compartmental choroidal analysis [34], and the integration, within a single temporal observation, of choroidal structure and vascularity, retinal SCP microcirculation, ONH perfusion and IOP. The a priori power analysis confirmed that the enrolled sample was more than adequate for the observed effects.

On the other hand, several limitations should be acknowledged. First, the cohort was restricted to young, healthy volunteers, which limits generalizability to eyes with ocular or systemic disease. Second, the observational, single-group design without a comparison arm does not allow the temporal changes to be attributed to caffeine with the same certainty as a controlled or crossover design would provide. Third, the observation window was short (1 h) and does not capture the later phases of the response (4–6 h) or possible rebound phenomena. Fourth, the deep capillary plexus was not analyzed, owing to well-recognized artifact and segmentation limitations that reduce the reliability of its density metrics [40,41]. Fifth, IOP was measured with non-contact tonometry rather than Goldmann applanation, which may reduce absolute precision while preserving relative consistency across the repeated measurements. Sixth, no biochemical or systemic parameters were assessed. Plasma caffeine concentrations (together with inter-individual differences in absorption and metabolism, which could be potentially modulated by CYP1A2 and ADORA2A genotype), systemic hemodynamic variables (blood pressure and heart rate), metabolic markers such as blood glucose, dietary history and physical activity were not measured; these could help disentangle local ocular effects from systemic influences and clarify the relationship between the observed ocular changes and systemic physiology. In particular, systemic factors such as glycemic variability and arterial blood pressure are known to influence choroidal thickness and retinal microvascular density, even in non-diabetic and normotensive individuals [42,43]. As these parameters were not recorded, we cannot fully exclude their contribution to the inter-individual variability of the response. However, the within-subject design over a short time window and the anatomical selectivity of the observed effect make it unlikely that these factors account for the progressive choroidal changes attributed to caffeine. Future multidisciplinary studies integrating ophthalmic imaging with biochemical and hemodynamic monitoring would provide a more comprehensive characterization of the systemic-ocular effects of caffeine. Seventh, choroidal thickness and the compartmental areas were obtained through manual segmentation performed by a single observer (E.D.). Although all measurements followed a standardized protocol under constant illumination and image-quality control to maximize intra-observer reproducibility, manual segmentation remains inherently operator-dependent, and the absence of a second grader precluded the assessment of inter-observer agreement. Automated or semi-automated segmentation algorithms, together with multi-observer designs, would help minimize subjectivity and further strengthen measurement reliability in future studies. Finally, although all participants abstained from caffeine for at least 24 h before testing, their habitual caffeine consumption was not recorded and could not be incorporated into the analysis. Considering that chronic intake may induce pharmacodynamic tolerance (through upregulation of adenosine receptors) and caffeine metabolism varies between individuals, habitual consumption represents a potential source of inter-individual variability in the magnitude of the acute response. Furthermore, as the present study assessed choroidal structural and vascularity parameters rather than directly measuring choroidal blood flow or vessel caliber, the adenosine-mediated vasoconstrictive mechanism proposed here should be regarded as a plausible interpretation rather than a directly demonstrated finding. Future crossover studies should record habitual consumption and, where possible, stratify participants (naïve, occasional, and habitual consumers), ideally combining plasma caffeine quantification and genotyping, to clarify the role of tolerance and to identify potential responders and non-responders.

## 5. Conclusions

A single 200 mg dose of caffeine in healthy young adults was associated with an acute reduction in SCT, accompanied by a concurrent, parallel decrease in LCA and SCA. As a result, the CVI shows only a minimal increase; the retinal SCP and the ONH microcirculation showed no significant short-term changes, while IOP showed a modest but significant reduction. This pattern could be consistent with a potential transient adenosine-mediated vasoconstrictive mechanism predominantly affecting the choroid.

The findings underline the need to standardize the timing of caffeine intake in choroid-driven studies and in clinical protocols that include OCT or OCTA measures sensitive to hemodynamic variation. Future studies should extend the observation to longer windows (4–6 h), adopt crossover designs with different doses and matrices, quantify plasma caffeine, integrate multi-plexus and peripapillary analyses, and address at-risk populations (glaucoma, pachychoroid disease, central serous chorioretinopathy, age-related macular degeneration, vascular retinopathies), ideally incorporating genetic (CYP1A2, ADORA2A) and behavioral covariates to distinguish acute from tolerance-modulated responses.

## Figures and Tables

**Table 1:** Descriptive statistics of all parameters at the three time points.

Parameter	Baseline	30 min	1 h	Δ%
**SCT (µm)**	356.0 ± 84.2	339.7 ± 82.3	325.9 ± 81.0	−8.5%
**TCA (mm^2^)**	1.048 ± 0.236	1.028 ± 0.232	0.995 ± 0.212	−5.1%
**SCA (mm^2^)**	0.390 ± 0.097	0.379 ± 0.099	0.366 ± 0.089	−6.0%
**LCA (mm^2^)**	0.659 ± 0.140	0.649 ± 0.134	0.629 ± 0.123	−4.6%
**CVI (%)**	63.01 ± 1.28	63.38 ± 1.71	63.43 ± 1.50	+0.7%
**IOP (mmHg)**	13.9 ± 2.2	13.1 ± 2.3	13.0 ± 2.7	−6.5%
**SCP vessel density (mm/mm^2^)**	15.7 ± 3.0	15.7 ± 2.8	16.2 ± 2.2	+3.2%
**SCP perfusion (%)**	38.5 ± 7.8	38.6 ± 7.1	39.6 ± 5.8	+2.8%
**ONH perfusion (%)**	44.7 ± 1.8	44.4 ± 1.9	44.5 ± 1.6	−0.6%
**ONH flow index**	0.429 ± 0.032	0.422 ± 0.034	0.426 ± 0.033	−0.7%

Abbreviations: SCT = subfoveal choroidal thickness; TCA = total choroidal area; LCA = luminal choroidal area; SCA = stromal choroidal area; CVI = choroidal vascularity index; IOP = intraocular pressure; SCP = superficial capillary plexus; ONH = optic nerve head; Δ = difference between 1 h and baseline.

**Table 2:** Repeated-measures ANOVA results for each parameter.

Parameter	F	*p*-Value	η^2^*p*	Sphericity	Friedman *p*
**SCT**	70.6	<0.001	0.61	GG corrected	<0.001
**TCA**	20.0	<0.001	0.31	GG corrected	<0.001
**SCA**	19.8	<0.001	0.31	GG corrected	<0.001
**LCA**	17.8	<0.001	0.28	GG corrected	<0.001
**CVI**	6.13	0.003	0.12	Preserved	0.003
**IOP**	5.52	0.009	0.11	GG corrected	0.036
**SCP vessel density**	0.90	0.411	0.02	Preserved	0.389
**SCP perfusion**	0.68	0.508	0.01	Preserved	0.544
**ONH perfusion**	1.21	0.303	0.03	Preserved	0.056
**ONH flow index**	1.31	0.275	0.03	Preserved	0.266

Abbreviations: SCT = subfoveal choroidal thickness; TCA = total choroidal area; LCA = luminal choroidal area; SCA = stromal choroidal area; CVI = choroidal vascularity index; IOP = intraocular pressure; SCP = superficial capillary plexus; ONH = optic nerve head. η^2^*p* = partial eta-squared; GG = Greenhouse–Geisser.

**Table 3:** Post hoc pairwise comparisons (Holm-corrected) for the significant parameters.

Parameter	Baseline vs. 30 min	Baseline vs. 1 h	30 min vs. 1 h
**SCT**	<0.001	<0.001	<0.001
**TCA**	0.001	<0.001	0.001
**SCA**	<0.001	<0.001	0.001
**LCA**	0.005	<0.001	0.001
**CVI**	0.036	0.006	0.653
**IOP**	0.014	0.036	0.857

Abbreviations: SCT = subfoveal choroidal thickness; TCA = total choroidal area; LCA = luminal choroidal area; SCA = stromal choroidal area; CVI = choroidal vascularity index; IOP = intraocular pressure.

## Data Availability

The data presented in this study are available on request from the corresponding author due to privacy restrictions, as the data contain sensitive clinical information of the participants.

## Abbreviations

The following abbreviations are used in this manuscript:

CSI	choroid–scleral interface
CVI	choroidal vascularity index
EDI	enhanced depth imaging
IOP	intraocular pressure
LCA	luminal choroidal area
OCT	optical coherence tomography
OCTA	OCT angiography
ONH	optic nerve head
RPE	retinal pigment epithelium
ROI	region of interest
SCA	stromal choroidal area
SCP	superficial capillary plexus
SCT	subfoveal choroidal thickness
TCA	total choroidal area
